# Development of Subject Specific Finite Element Models of the Mouse Knee Joint for Preclinical Applications

**DOI:** 10.3389/fbioe.2020.558815

**Published:** 2020-10-15

**Authors:** Sahand Zanjani-Pour, Mario Giorgi, Enrico Dall'Ara

**Affiliations:** ^1^Department of Oncology and Metabolism, Mellanby Center for Bone Research, University of Sheffield, Sheffield, United Kingdom; ^2^Insigneo Institute for in Silico Medicine, University of Sheffield, Sheffield, United Kingdom; ^3^Certara Quantitative System Pharmacology, Certara UK Ltd., Simcyp Division, Sheffield, United Kingdom

**Keywords:** mouse knee, finite element, subject specific, PTA staining, cartilage, bone

## Abstract

Osteoarthritis is the most common musculoskeletal disabling disease worldwide. Preclinical studies on mice are commonly performed to test new interventions. Finite element (FE) models can be used to study joint mechanics, but usually simplified geometries are used. The aim of this project was to create a realistic subject specific FE model of the mouse knee joint for the assessment of joint mechanical properties. Four different FE models of a C57Bl/6 female mouse knee joint were created based on micro-computed tomography images of specimens stained with phosphotungstic acid in order to include different features: individual cartilage layers with meniscus, individual cartilage layers without meniscus, homogeneous cartilage layers with two different thickness values, and homogeneous cartilage with same thickness for both condyles. They were all analyzed under compressive displacement and the cartilage contact pressure was compared at 0.3 N reaction force. Peak contact pressure in the femur cartilage was 25% lower in the model with subject specific cartilage compared to the simpler model with homogeneous cartilage. A much more homogeneous pressure distribution across the joint was observed in the model with meniscus, with cartilage peak pressure 5–34% lower in the two condyles compared to that with individual cartilage layers. In conclusion, modeling the meniscus and individual cartilage was found to affect the pressure distribution in the mouse knee joint under compressive load and should be included in realistic models for assessing the effect of interventions preclinically.

## Introduction

Osteoarthritis (OA) is highly prevalent in our aging society (Wallace et al., 2017) and is the most common degenerative joint disorder, affecting 8.5 million adults in the UK (Neogi, 2014) and costing the economy more than 4.2 billion pounds (Chen et al., 2012). Currently, there are no pharmacological treatments available for OA (Anandacoomarasamy and March, 2010; Hermann and Muller-Ladner, 2018) and patients have to undergo invasive total joint replacement surgeries to reduce the pain and regain mobility (Kremers et al., 2014; de l'Escalopier et al., 2016). Therefore, there is a need for pre-clinical assessment of novel interventions in animal models.

The mouse is one of the most used animals in OA research (Christiansen et al., 2015; Kuyinu et al., 2016) thanks to the relative low costs associated to the *in vivo* studies and the possibility of creating OA disease phenotypes through genetic modification (Zhang et al., 2014) or destabilization of the joint resecting portion of the meniscus (DMM) (Glasson et al., 2007; Culley et al., 2015) or of the ligaments (Clements et al., 2003).

**Figure 1:**
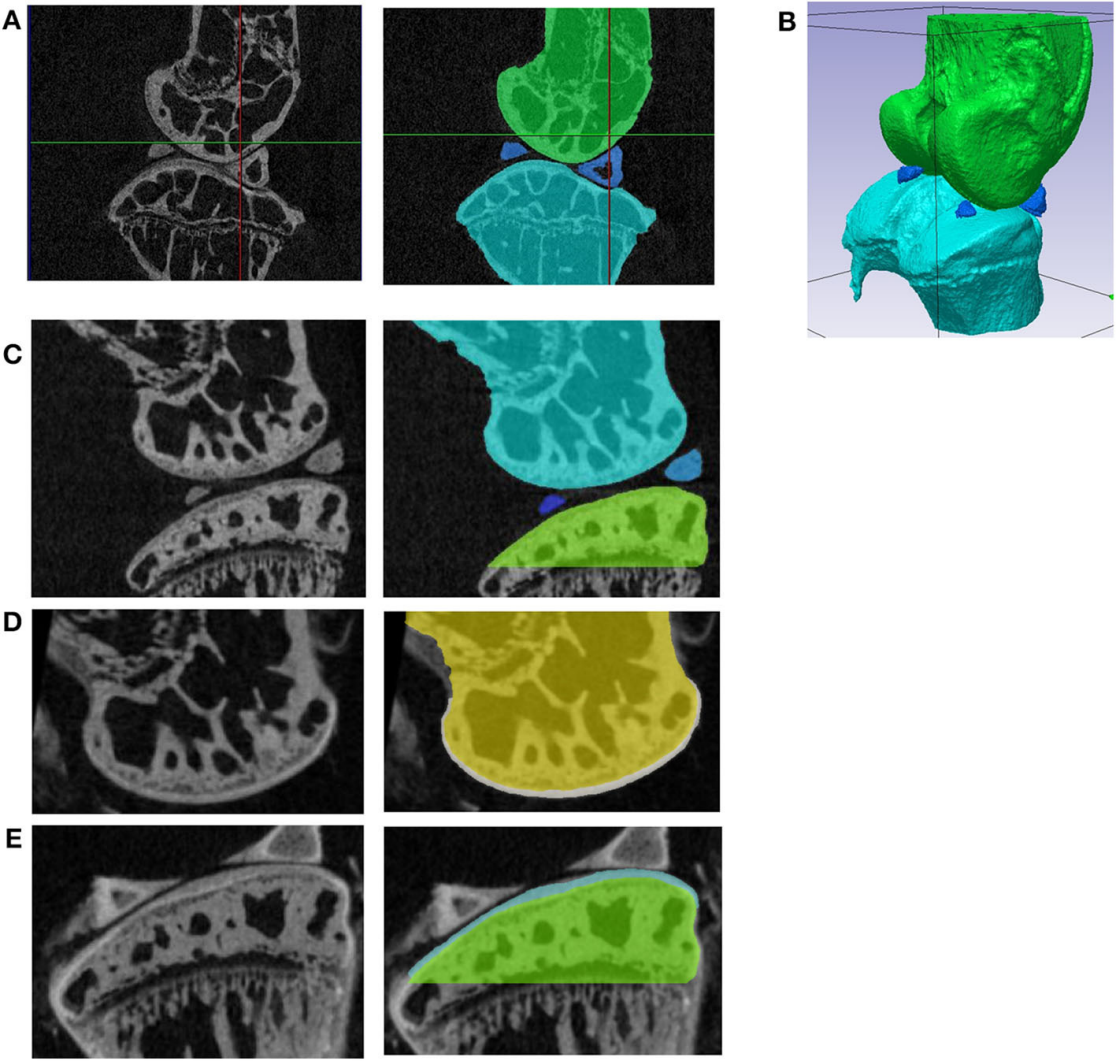
**(A)** Sagittal section of the microCT image of the unstained specimen with the joint in the rest position; segmentation of the femur (green), tibia (light blue), and calcified menisci (dark blue); **(B)** 3D view of the segmented bones in the resting position; **(C)** sagittal section from the microCT image acquired before staining (left) and segmentation of the bone tissue (right). The images of the femur **(D)** and tibia **(E)** from the microCT scans acquired after staining (left) were registered to the images in **(A)** and the cartilage was segmented (right). In **(E)** portions of the menisci that could not be removed during the dissection without risk of damaging the cartilage are visible.

Realistic finite element (FE) models of the whole mouse joint would be helpful to study the effect of interventions and treatments on the mechanical properties of the cartilage (Silva et al., 2005; Das Neves Borges et al., 2014; Yang et al., 2014). However, creating realistic geometries is challenging due to the complex anatomy of the mouse knee (Charles et al., 2016, 2018), that consists of bones (distal femur and proximal tibia) and soft tissues, including a meniscus with calcifications within its structure (example in Figure 1). Subject specific geometry of the bone in the knee joint can be acquired by imaging techniques such as *in vivo* micro computed tomography (microCT) (Dall'Ara et al., 2016), *ex vivo* microCT (Das Neves Borges et al., 2014) or Synchrotron radiation microCT (Madi et al., 2019). However, to study the effect of OA and related interventions on the joint morphology and biomechanics, also soft tissues should be studied in details (Das Neves Borges et al., 2014). Nevertheless, soft tissues as cartilage and menisci are not visible in microCT scans of unstained specimens. A recent study showed that high-resolution systems and phase contrast can be used to visualize the cartilage geometry of osteochondral plugs without staining (Clark et al., 2019). Nevertheless, this approach has not been validated on whole joints of rodents yet. In previous studies, phosphotungstic acid (PTA) staining was used to visualize cartilage in microCT images of the mouse knee joint (Marenzana et al., 2012, 2014; Das Neves Borges et al., 2014, 2017) and the obtained geometry of the cartilage was included in subject specific FE models (Das Neves Borges et al., 2014). However, the FE models did not include the menisci, which was found to play a critical role in the joint mechanics (Ramos-Mucci et al., 2020).

The aim of this study was to define a workflow to create realistic subject specific FE models of the mouse knee joint including cartilage and menisci for the estimation of the bone and cartilage mechanical properties under compressive loading. The importance of individual geometries of the soft tissues when modeling the mouse knee joint was evaluated by comparing the outputs of realistic and simplified models.

## Materials and Methods

The tissues used in this study were collected from previous animal work, performed under a British Home Office project license (PPL 40/3499) and in compliance with the UK Animals (Scientific Procedures) Act 1986. The right hind limb of a female, 16 weeks old C57BL/6 mouse was dissected from a freshly culled mouse (IV overdose injection). After removing the skin, the limb was fixed in the buffered formalin and then stored in ethanol 70%.

### Bone Geometry From Rest Position Scan of Unstained Specimens

The hind limb, with all the remaining soft tissues, was mounted in polystyrene and scanned in rest position with a microCT (Skyscan1172, Bruker) using the following scanning parameters: intensity 200 μA; voltage 50 kV; beam hardening filer Al 0.5 mm; isotropic voxel size 4.35 μm; rotation step 0.7°, 180° scan. The image was then reconstructed (NRecon, Skyscan) with the following parameters: ring artifact correction = 10, beam hardening correction = 30%, dynamic range = 0–0.13 (Figure 1A).

The proximal tibia, distal femur and calcified meniscus (visible in healthy mice knee joints) were cropped from the images, smoothed (Gaussian, Sigma = 1 voxel) and segmented (single level threshold based on histograms, Simpleware ScanIP, Synopsys, USA, Figure 1B).

### Cartilage Geometry From Separated Scans of Stained Specimens

After the rest position scan the specimen was further dissected under a dissection microscope and the tibia and femur were separated, removing most of the remaining soft tissue except the cartilage. The tibia and the femur were incubated for 24 hours on a rocking platform in a solution of PTA 1% and ethanol 70% (1 unit PTA solution and 2 units ethanol solution) (Marenzana et al., 2014).

The tibia and femur were positioned in a sample holder filled with ethanol 70% with the distal portion of the femur and the proximal portion of the tibia facing each other similarly to the resting position but leaving a larger gap between the bones to facilitate the segmentation of the cartilage. The distal tibia and proximal femur were glued to the holder to reduce moving artifacts. Another microCT image was acquired with the same scanning parameters used for the scan performed in the rest position, except the rotation step that was reduced from 0.7 to 0.3° to increase the image quality. The same reconstruction parameters were used.

After cropping the images of the femur and tibia, each bone was independently rigidly registered to the images obtained in rest position using a landmark registration in two steps. First a rough registration with three markers (Amira v6, Thermo Fisher Scientific) was applied in order to select similar regions of interest and reduce the image size, then the images were imported into Scan IP (Simpleware Synopsis) and registered with 15 landmarks chosen in features of the bone tissue identified in both images (“background registration” function, linear interpolation).

Due to the low contrast between the cartilage and the bone in the microCT images after staining, single level threshold was not satisfactory enough for cartilage segmentation. Therefore, the contours of cartilage tissue were manually identified in every slice, in the transverse plane. 3D masks were generated (“flood fill” tool) and adjusted in coronal and sagittal sections. After smoothing (2-pixel radius) the original bone masks (Figure 1C) were subtracted using Boolean operations, leaving tibia and femur cartilage layers on top of the bones (Figures 1D,E).

### Geometry of Meniscus

**Figure 2:**
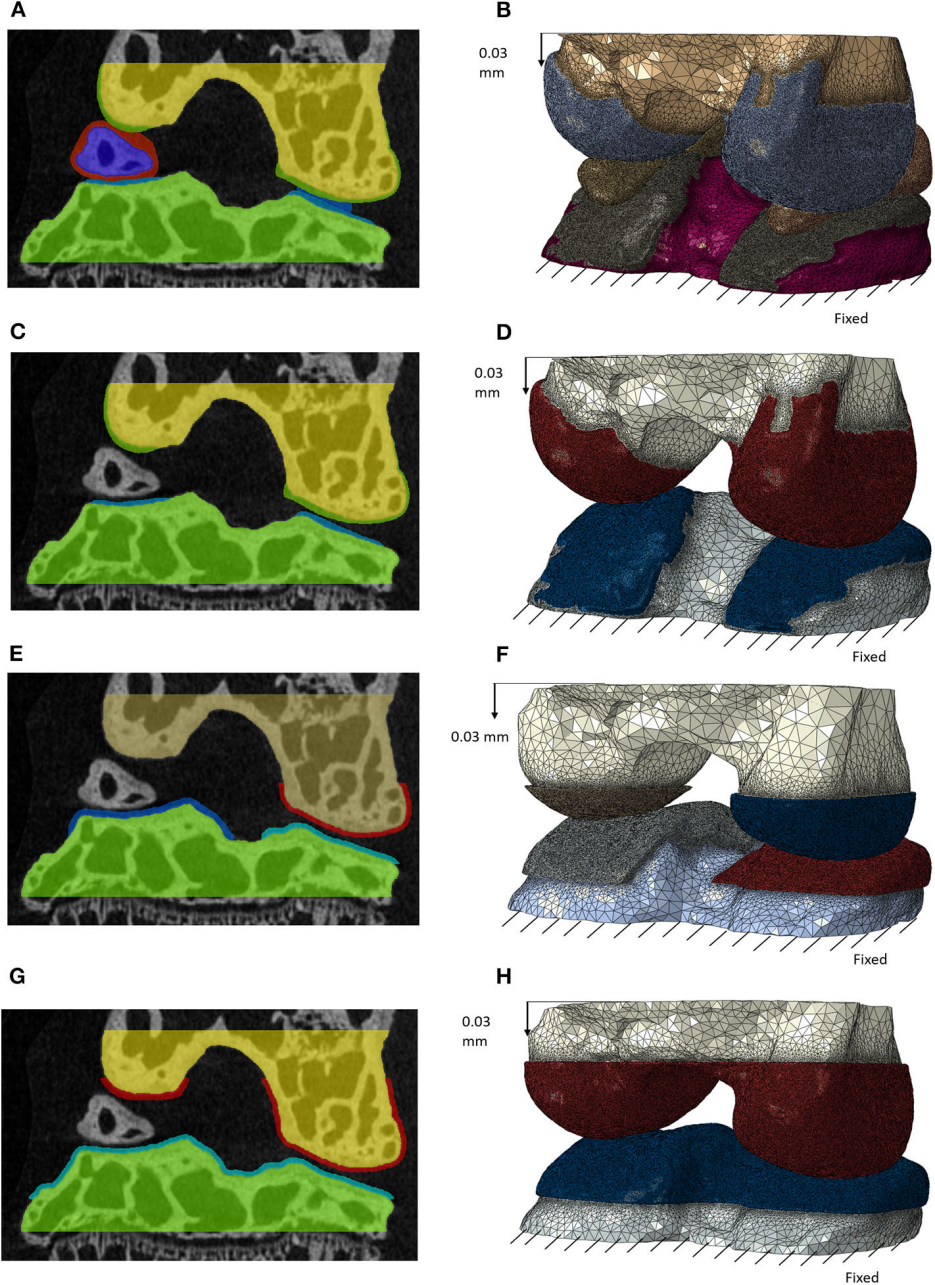
Masks of the features for the different models with decreasing complexity in frontal section (left) and the generated finite element models after meshing (right) for: model including individual cartilage layers with meniscus **(A,B)**; model including individual cartilage layers without meniscus **(C,D)**; model including homogeneous cartilage layers with different thickness values for the lateral and medial condyles **(E,F)**; model including homogeneous cartilage layer with the same value of thickness for both condyles **(G,H)**. In image **(E)** the cartilage is not visible for the medial condyle due to the chosen cutting plane. A supplementary image is included to show this layer (Supplementary Figure 1).

The calcified portions of the meniscus, typical anatomical features in mice, were segmented from the rest position scan (Figures 1A,B). The soft tissues of the menisci, not visible in either of the two microCT scans, were created by filling the gap between the calcified menisci and the external surface of the cartilage of the tibia and of the femur (function “3D wrap” in Simpleware Scan IP). This simplification was unavoidable due to the lack of information about the meniscus structure in the microCT images. After 3D wrapping the meniscus mask had multiple intersections with other existing masks. These intersections in meniscus masks were deleted using Boolean operations. Furthermore, the portions of the meniscus around its calcifications and between the condyles were manually edited to fit in the anatomy as shown in Figure 2A.

### Generic Cartilage Geometries

Two models with generalized cartilage were created. In the first model one layer of cartilage with the same thickness over both condyles was created so that the two cartilage layers of the lateral condyle (the one with the lower gap between the cartilage surfaces) were in contact (Figure 2G). In the second, two layers of cartilage with different thickness were created in the two condyles, so that the cartilage layers of tibia and femur were in contact for both condyles (Figure 2E).

### Finite Element Models

Four models were generated from the above-described geometries:

1. Individual model including the meniscus, individual cartilage layers and bones (Figure 2B);
2. Individual model without meniscus, including the individual cartilage layers and bones (Figure 2D);
3. Model with two generic cartilage layers with different thickness for medial and lateral condyles and bones (Figure 2F);
4. Model with one generic cartilage layer with the same thickness for both condyles and bones (Figure 2H).

For all models, the femur was moved 87 μm (20 voxels) in the superior direction to create a gap between the touching cartilage layers and to simplify the meshing and initialization of the contact. The bone and soft tissue geometries were meshed with linear tetrahedral elements (Simpleware scan IP, +FE Free Function) (Das Neves Borges et al., 2014) with target minimum edge length of 100 μm for bones and 5 μm for soft tissues (-9 coarseness in Simpleware Scan IP). Considering that thickness of the subchondral plate (cortical bone and calcified cartilage) was high and homogenous across the condyles, the bone was modeled as homogeneous isotropic material. Linear homogenous isotropic material properties were assigned to each element [Cartilage: E = 6 MPa, ν = 0.49; Meniscus: E = 59 MPa, ν = 0.49; and bone: E = 18000 MPa, ν = 0.3 (Peña et al., 2005; Das Neves Borges et al., 2014)].

The meshes were imported in Abaqus/CAE (Dassault Systemes, USA). For all models, the nodes of the most distal portion of the tibia were fixed in every Cartesian direction. The superior node set of the femur was dynamically coupled to a reference point chosen in the center of the top surface of the femur. Considering that the ligaments were not modeled, simulations in displacement control were performed, assuming that the ligaments and the other not modeled soft tissues would keep in position the joint during loading. An axial displacement of 87 μm was applied to the reference point to initialize the contact between the femur cartilage and the meniscus or tibia cartilage. Afterwards, an axial displacement of 30 μm was applied to the reference point in four steps (Figures 2B–H).

The interaction between the cartilage of the femur (master surface) and the cartilage of the tibia or the soft meniscus (slave surfaces) were modeled as surface-to-surface with finite sliding, frictionless tangential contact, normal hard contact with default penalty stiffness. In the case of the individual model with meniscus, perfect bonding was assumed between the distal nodes of the soft meniscus and the distal surface of the cartilage of the tibia.

The results between the different models were compared at the loading step that provided the closest value of reaction force of 0.3 N. Strain and stress distributions in the bone and soft tissue and cartilage contact pressure were analyzed and compared for the four models. Frequency plots for the cartilage pressure were created for each condyle for each model and peak cartilage pressure were defined excluding isolated points, due probably to local geometrical artifacts. Average values of contact pressure between the femoral and tibial cartilage layers were also calculated for each condyle.

## Results

The models were solved with ShARC HPC Cluster available nodes (Dell PowerEdge C6320, 2 x Intel Xeon, 64 GB). The individual model with meniscus took ~24 h and the other models ~12 h.

**Figure 3:**
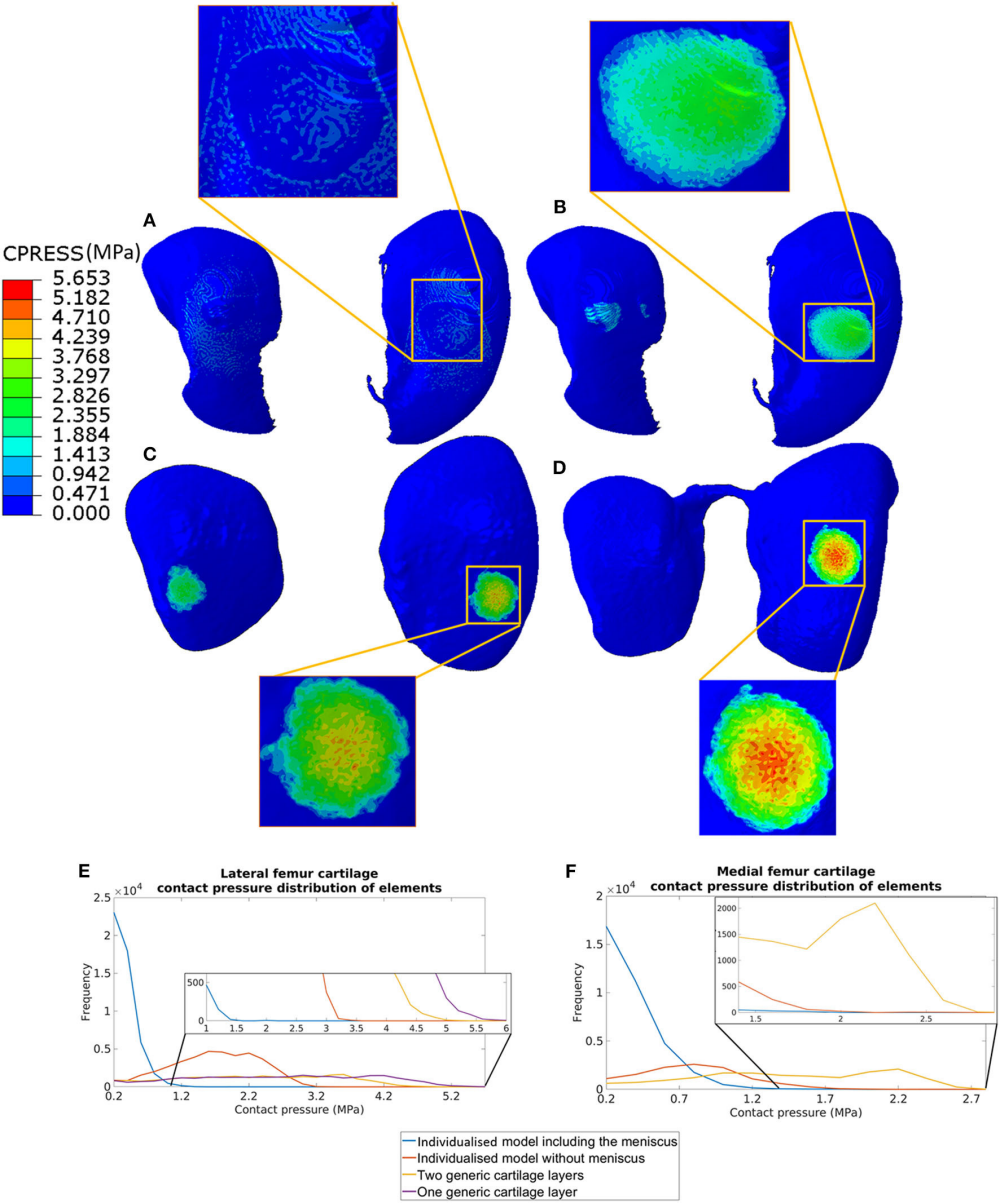
Contact pressure distribution in the femoral cartilage for the four models at 0.3 N reaction force: **(A)** model including individual cartilage layers with meniscus; **(B)** model including individual cartilage layers without meniscus; **(C)** model including homogeneous cartilage layers with different thickness values for the lateral and medial condyles; **(D)** model including homogeneous cartilage layer with the same value of thickness for both condyles. The distribution of contract pressure for lateral **(E)** and medial **(F)** condyle cartilages are reported. The contact pressure for medial femur cartilage in the model with homogeneous cartilage layer with the same thickness is zero as there are no contacts between the cartilage of the femur and of the tibia.

**Figure 4:**
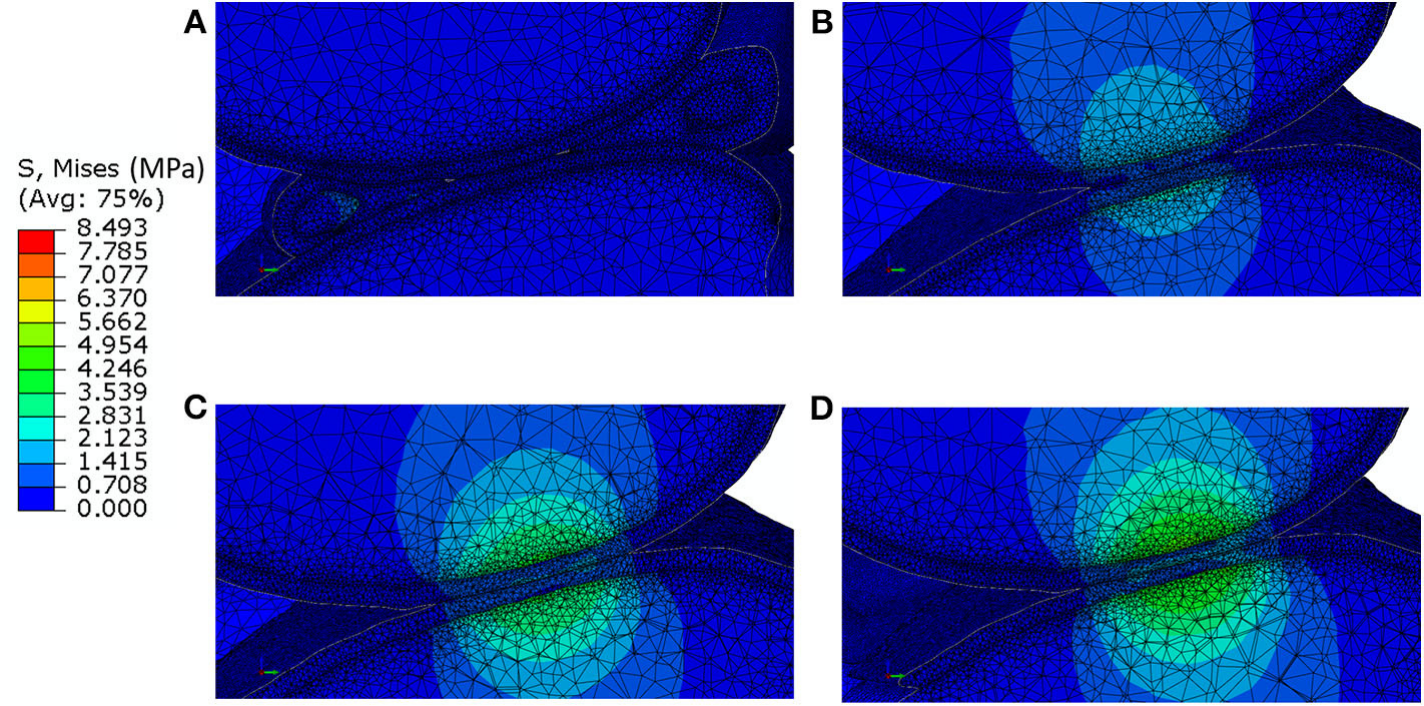
Von-Mises stress distribution at 0.3 N reaction force in a sagittal section passing through the lateral condyle and containing the point of contact between the femoral cartilage and the tibia cartilage for: **(A)** model including individual cartilage layers with meniscus; **(B)** model including individual cartilage layers without meniscus; **(C)** model including homogeneous cartilage layers with different thickness values for the lateral and medial condyles; **(D)** model including homogeneous cartilage layer with the same value of thickness for both condyles. It should be noted that the stress distribution in the bone does not take into account for the microstructural properties of the bone in that area.

The distribution of contact pressure in the elements of the distal surface of the femoral cartilage at ~0.3 N reaction force are reported for the four models in Figures 3A–D. The pressure distribution was much more concentrated and the peak values much higher in the generic models compared to those with subject-specific cartilage. Furthermore, modeling the meniscus lead to a much more homogeneous pressure distribution across a larger area of the condyles (Figures 3A–D). The highest average and peak contact pressure in the lateral femoral cartilage was found for the model with one layer of generalized cartilage (average: 2.7 MPa; peak: 5.6 MPa), followed by the model with two layers of cartilage with different thickness values (average: 2.3 MPa, −15% compared to the simplest model; peak: 5.0 MPa, −11% compared to the simplest model), by the model with individual cartilage (average 1.7 MPa, −37% compared to the simplest model; peak: 3.2 MPa, −43% compared to the simplest model), and by the model with individual cartilage and meniscus (average: 0.4 MPa, −85% compared to the simplest model; peak: 2.1 MPa, −63% compared to the simplest model, Figures 3E,F). The model with individual meniscus showed a much larger number of elements with low contact pressure between 0.2 and 1.0 MPa (Figures 3E,F). For the medial condyles, there was no contact in the homogenous cartilage model. The highest average and peak contact pressure in the medial femoral cartilage was found for the model with two thickness layers of generalized cartilage (average: 1.5 MPa; peak: 2.8 MPa), followed by the model with individual cartilage (average: 0.8 MPa, −47% compared to the model with two layers of cartilage thickness; peak: 2.0 MPa, −29% compared to the model with two layers of cartilage thickness), and by the model with individual cartilage and meniscus (average: 0.2 MPa, −87% compared to the model with two layers of cartilage thickness; peak: 1.9 MPa, −32 % compared to the model with two layers of cartilage thickness). The distributions of contact pressure in the femoral cartilage were consistent with the Von-Mises stress distributions as observed from the sagittal section containing the middle of the lateral condyle (Figure 4).

## Discussion

The aim of this study was to investigate the importance of modeling subject-specificity features of the mouse knee joint for computing the cartilage contact pressure distribution, which is linked to the tissue degeneration and fundamental to study preclinically the effect of induced post-traumatic OA and related interventions.

The most complex model used in this study included for the first time the meniscus and realistic layers of cartilage obtained from microCT images of stained soft tissues. Moreover, the models were generated from images acquired in the rest position of the mouse hindlimb, in order to improve the boundary conditions in the FE models. The results showed that the cartilage contact pressure changed dramatically from the model with or without subject-specific features. The results of the generated models showed a decrease of ~1 MPa in the average and peak contact pressure in both condyles (25% lower in medial condyle and 27% lower in the lateral condyle, Figures 3A–D) and a shift in the distribution of the contact pressure toward lower values in the models with realistic cartilage geometry (Figures 3E,F). The decrease in cartilage pressure, due to a larger region of the tissue in contact under the considered load in the models with subject-specific cartilage, highlights the importance of accounting for realistic cartilage geometry when estimating the contact pressure distribution and linking it to the degeneration of the cartilage (Guilak, 2011; Buckwalter et al., 2013). In a similar study by Das Neves Borges et al. (2014), the peak contact pressure was not reported as the aim of that study was to compare the pressure distribution among Naïve, Control and DMM mice. Nevertheless, from the reported figures it seems that the peak in contact pressure in the femur at 0.6 N load was in the range of 6–8 MPa. While similar distributions between their cartilage pressure and the one obtained with individual cartilage in this study were observed, the higher pressure peaks found in that study can only be partially explained by the higher load used in the simulations (0.3 N in or case). This difference can be due to the different segmentation procedure and the position of the joint in the simulation. In particular, in this study the cartilage was segmented manually and the initial relative position between the femur and tibia was based on a scan performed in the rest position. Conversely, in Das Neves Borges' study (Das Neves Borges et al., 2014), automatic segmentation was performed and the femur was placed 80° flexion with regards to the tibia.

The importance of the role of meniscus in mouse knee joint has been discussed in the literature (Ramos-Mucci et al., 2020). In this study, the impact of including the meniscus in the mouse knee joint models has been investigated by comparing the two models with and without meniscus (with realistic cartilage). Modeling the meniscus affected the values and distribution of the cartilage contact pressure. The peak cartilage contact pressure was reduced by 34% in the lateral condyle and by 5% in the medial condyle (Figure 3). The distribution of the pressure in the two condyles was much more homogeneous in the model with the menisci, highlighting its function of re-distributing the load on a large surface of the cartilage, which probably leads to a reduction in joint degeneration over time. These results are particularly important for applications of these FE models in studying the effect of interventions as the destabilization of the medial meniscus (DMM), typical mouse model for post-traumatic OA, on the knee contact pressure (Glasson et al., 2007; Culley et al., 2015). In these cases, the models with meniscus and without meniscus (both with realistic cartilage) should be used for the evaluation of the effect of the destabilization of the joint on the contact pressure distribution. Similarly, these models can be used to study *in silico* the effect of different post-traumatic OA animal model or of treatments that affect the cartilage and/or bone geometry and material properties.

This study has three main limitations. The study was performed only on one specimen and it should be generalized by including analyses of joints from different ages and with or without interventions. Nevertheless, the study was focused on the development of the approach. Furthermore, the considered material properties are simplified to isotropic linear elastic. While this approach is reasonable for comparing models with geometrical differences, in the future in order to obtain realistic stress and strain distributions more complex material properties accounting for poroelastic and viscoelastic properties of cartilage should be assigned (Mononen et al., 2012; Kazemi and Li, 2014; Kłodowski et al., 2016; Stender et al., 2017). Moreover, the geometry of the meniscus was simplified in this study, due to the lack of details observable in the microCT images. In order to model the meniscus more realistically mouse-specific geometries should be included and more complex material properties should be assigned (e.g., hyper-elastic fiber reinforced material). Other imaging approaches using phase contrast imaging (Clark et al., 2019) or Synchrotron based tomography (Madi et al., 2019), could be used to improve the definition of the geometry and microstructure of the modeled features, which could also be used to improve the constitutive models. Finally, tendons and ligaments were not included in the current model and should be added for more realistic joint models and in case load-controlled simulations are developed.

In conclusion, this study highlights the important of modeling the meniscus and realistic geometry of the cartilage layers for the estimation of contact pressure in mouse knee finite element models. In the future, this model can be used to study the effect of induced OA and related treatments in pre-clinical mouse models.

## Data Availability Statement

The raw data supporting the conclusions of this article will be made available by the authors, without undue reservation. The data can be found at: doi: 10.15131/shef.data.12924920.

## Ethics Statement

The animal study was reviewed and approved by the tissues used in this study were collected from previous animal work, performed under a British Home Office project license (PPL 40/3499) and in compliance with the UK Animals (Scientific Procedures) Act 1986.

## Author Contributions

SZ-P: data acquisition and drafting. ED'A and SZ-P: analysis and interpretation of the data. ED'A and MG: revision of the article. All authors have been involved in the conception, design of the study, reviewed, and agreed upon the last version of the manuscript.

## Conflict of Interest

MG was employed by the company Certara UK Ltd. Certara QSP provided support in the form of salaries for authors during the writing of the manuscript (MG), but did not have any additional role in the study design, data collection and analysis, decision to publish, or preparation of the manuscript. The remaining authors declare that the research was conducted in the absence of any commercial or financial relationships that could be construed as a potential conflict of interest.
