# Supplementary material for: Development of Subject Specific Finite Element Models of the Mouse Knee Joint for Preclinical Applications
Source: Front Bioeng Biotechnol. 2020 Oct 15;8:558815. doi: 10.3389/fbioe.2020.558815 (PMC7593650; doi:10.3389/fbioe.2020.558815)
Supplement: Supplementary file 1 [file Table_1.DOCX]

SUPPLEMENTARY MATERIAL

**
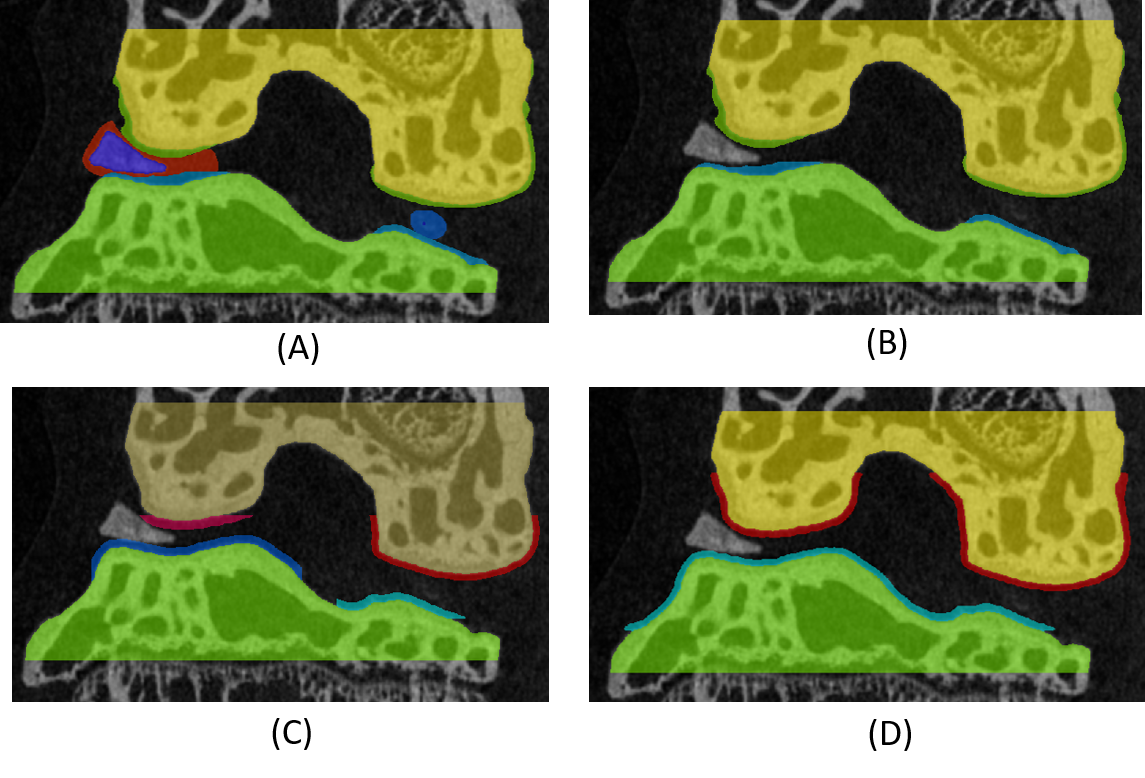
**

Supp Figure 1: Masks of the features for the different models with decreasing complexity: (A) Individual cartilage with meniscus with meniscus; (B) individual cartilage without meniscus; (C) homogeneous cartilage layers with different thickness values; (D) homogeneous cartilage layer. Another cut plane was considered in this figure compared to Figure 3 in order to show the two different cartilage layers in (C).
